# Clinical significance of palliative gastrectomy on the survival of patients with incurable advanced gastric cancer: a systematic review and meta-analysis

**DOI:** 10.1186/1471-2407-13-577

**Published:** 2013-12-05

**Authors:** Jingxu Sun, Yongxi Song, Zhenning Wang, Xiaowan Chen, Peng Gao, Yingying Xu, Baosen Zhou, Huimian Xu

**Affiliations:** 1 Department of Surgical Oncology and General Surgery, First Hospital of China Medical University, Shenyang 110001, China; 2 Department of Epidemiology, School of Public Health, China Medical University, Shenyang 110001, China

**Keywords:** Gastric cancer, Incurable, Palliative gastrectomy, Metastasis, Meta-analysis

## Abstract

**Background:**

Palliative gastrectomy for patients with advanced gastric cancer remains controversial. The objective of the present meta-analysis was to analyze survival outcomes and establish a consensus on whether palliative gastrectomy is suitable for patients with incurable advanced gastric cancer and which type of patients should be selected to receive palliative gastrectomy.

**Methods:**

A literature search was conducted in PubMed, EMBASE and the Cochrane Library. The results for overall survival in the meta-analysis are expressed as hazard ratios (HRs) with 95% confidence intervals (CIs).

**Results:**

Of 1647 articles and abstracts reviewed, 14 studies with 3003 patients were eligible for the final analysis. The meta-analysis revealed that palliative gastrectomy is associated with a significantly improvement in overall survival (HR 0.56; 95%CI 0.39–0.80; p < 0.002) compared that of patients treated without palliative gastrectomy. An improvement in survival was also observed in patients with stage M1 gastric cancer who received palliative gastrectomy (HR 0.62; 95%CI 0.49–0.78; p < 0.0001), especially those with peritoneal dissemination (HR = 0.76, 95%CI 0.63–0.92), liver metastasis (HR = 0.41, 95%CI 0.30–0.55), or distant lymph-node metastasis (HR = 0.36, 95%CI 0.23–0.59). Combined hepatic resection may be beneficial for patients who under palliative gastrectomy (HR 0.30; 95%CI 0.15–0.61; p = 0.0008). The overall survival of patients who underwent palliative gastrectomy combined with chemotherapy was significantly improved (HR 0.63; 95%CI 0.47–0.84; p = 0.002).

**Conclusions:**

From the results of the meta-analysis, palliative gastrectomy for patients with incurable advanced gastric cancer may be associated with longer survival, especially for patients with stage M1 gastric cancer. Combined hepatic resection for patients with liver metastasis and chemotherapy may be beneficial factors compared to simple palliative gastrectomy.

## Background

In spite of significant advances in experimental research, diagnosis and treatment, gastric cancer (GC) accounts for over 10% of cancer-related deaths worldwide and remains the second most frequent cause of cancer death after lung cancer [1,2]. In recent years, however, the advances in new treatments and chemotherapy have improved the overall survival rate for GC patients with incurable factors compared with that of patients who receive only supportive treatment [3-5]. The long-term outcomes for early GC are improved with earlier diagnosis, but for advanced GC combined with incurable factors the results are not optimistic [6,7]. The incurable factors in patients with advanced GC are peritoneal dissemination, liver dissemination, distant lymph node metastases and a primary tumor of huge mass [8]. Therefore, palliative strategies are still necessary for patients with GC, especially in late stages [9].

The National Comprehensive Cancer Network (NCCN) guidelines suggest that gastric resections should be reserved for the palliation of symptoms (e.g., obstruction or uncontrollable bleeding) in patients with incurable disease [10]. The Japanese Gastric Cancer Association (JGCA) guidelines suggest that patients with metastases but without major symptoms may be treated with gastrectomy [11]. However, surgical resection is still considered to be the most suitable treatment for GC, but surgical resection for GC with incurable factors remains debatable. Palliative gastric resection could enable oral food intake, and decrease symptoms such as obstruction and bleeding [12,13]. Some investigations reported that gastric resection may be beneficial for survival, reducing symptoms, and enhancing the quality of life [13-17]. Simultaneously, some other studies reported that survival after palliative gastrectomy was associated with significant morbidity, longer hospital stays, and poor quality of life [18,19], and gastrectomy was recommended only for cases with serious complications, such as tumor bleeding or organ perforation [20,21].

Although many investigations have reported palliative gastrectomy for patients with incurable advanced GC, there is still not a clear consensus on the most suitable surgical treatment strategy. Also, determining which patients should receive palliative gastrectomy is also a question. Therefore, the present systematic review and meta-analysis was designed to analyze results according to surgical resection and factors that affect the survival of patients with incurable GC. The aim of our study was to determine the clinical significance of palliative gastrectomy for patients with incurable advanced GC focusing on patient selection and strategy selection.

## Methods

### Systematic search strategy

A sensitive search strategy was developed for all English language literature published before May 2013. The comprehensive search was performed using the electronic databases PubMed, EMBASE, and the Cochrane Library. The search strategy included the keywords “palliative gastrectomy”, “gastric cancer”, and “stomach neoplasm”, and the strategy was changed according to different requirements for each database. Review articles and bibliographies of other relevant identified investigations were hand-searched to identify additional studies. The articles were searched by two independent reviewers (Jingxu Sun and Xiaowan Chen), with any disagreements resolved by discussion and consensus. A list of titles and abstracts of potentially relevant studies were generated and imported in-to managerial software (EndNote®).

### Inclusion and exclusion criteria

All the studies included were comparative studies of patients with incurable advanced GC who received or did not receive palliative gastrectomy. Advanced GC was defined as T4N1–3 M0, T1–4N3M0, and any T or N with an M1 tumor category according to the TNM classification [22,23]. A total sample size of ≥50 patients was required and the procedure-related median survival, overall survival or survival curves were required to be reported. The articles that did not use the TNM staging system but included patients that were diagnosed with GC with metastasis were also included in the present study. Only published studies in peer-reviewed journals were included. Articles without full-text and data that could not be acquired from the authors were excluded. When multiple investigations were reported by the same team from the same institute done at the same time, only the latest or the article with the largest data set was included in the present study. Any useful supplemental data were also included if necessary.

### Data extraction and quality assessment of the included literature

Data collection and analyses were performed by two researchers using predefined tables, which included author, publication time, sample size, metastasis situation, chemotherapy situation, median survival time and overall survival. If the article did not provide the HR for overall survival, the software (Engauge Digitizer 4.1) was used to distinguish the survival curves and calculate the HRs of overall survival. The first reviewer (Sun JX) extracted the data and another reviewer (Chen XW) checked the data extraction.

A quality assessment of observational studies comparing patients with palliative gastrectomy and patients without palliative gastrectomy was performed using the Strengthening the Reporting of Observational Studies in Epidemiology (STROBE) guidelines (Table 1) [24]. Each item was described with Yes, No, or Partially.

**Table 1:** Quality assessment of trials included in the present study (STROBE)

**Author**	**Treatment**	**A**	**B**	**C**	**D**	**E**	**F**	**G**	**H**	**I**
Kikuchi S [26]	Palliative gastrectomy/other procedures	P	Y	N	Y	Y	Y	Y	N	Y
Saidi RF [27]	Palliative gastrectomy/no surgery	Y	Y	P	P	Y	N	Y	N	P
Nazli O [28]	Palliative gastrectomy/no surgery	P	Y	N	N	Y	N	Y	N	N
Lin SZ [29]	Palliative gastrectomy/unresectable operation/no surgery	Y	Y	N	P	Y	N	Y	N	N
Lupascu C [30]	Palliative gastrectomy/no surgery	Y	P	N	N	Y	N	Y	Y	P
Zhang JZ [31]	Palliative gastrectomy/no surgery	Y	P	N	Y	Y	N	Y	N	Y
Sougioultzis S [32]	Palliative gastrectomy/no surgery	Y	Y	N	Y	Y	N	Y	N	P
Kim KH [33]	Palliative gastrectomy/no surgery	Y	Y	N	Y	Y	Y	Y	N	Y
Chang YR [34]	Palliative gastrectomy/no surgery	Y	Y	N	Y	Y	Y	Y	N	Y
Kokkola A [35]	Palliative gastrectomy/exploration	Y	P	N	P	Y	P	Y	N	Y
Chen S [36]	Palliative gastrectomy/no surgery	P	Y	N	N	Y	N	Y	N	P
Tokunaga M [37]	Palliative gastrectomy/no surgery	Y	Y	P	Y	Y	N	Y	N	Y
Miki Y [38]	Palliative gastrectomy/no surgery	P	Y	N	Y	Y	N	Y	N	Y
Dittmar Y [39]	Palliative gastrectomy/unresectable operation/other procedures/no surgery	Y	Y	P	Y	Y	N	Y	N	N

**A**, Objectives and prespecified hypothesis in the introduction; **B**, Eligibility criteria of cohort in methods; **C**, Methods for recruitment of participant; **D**, Mention of outcomes, exposure, and confounder; **E**, Study size calculated; **F**, Potential biases addressed; **G**, Statistical methods described; **H**, Mention of how missing data was handled; **I**, Limitation of the study and the generalizations mentioned; Y, Yes; N, No; P, Partially.

### Statistics

The meta-analysis was performed with the Stata 12.0 and Review Manage Version 5.2 (RevMan 5.2) software and Microsoft Excel 2010 was used for the statistical analysis. The hazard ratio (HRs) and 95% confidence intervals (95%CIs) for the available data were calculated to identify potential associations with overall survival in the two groups, using the method reported by Tierney et al. [25]. Statistical heterogeneity across studies was quantified using the *χ*^2^ (or Cochran *Q* statistic) and *I*^2^ statistic. The *I*^2^ statistic is derived from the *Q* statistic ([*Q-df/Q*] × 100) and provides a measure of the proportion of the overall variation attributable to heterogeneity between the studies. If the test of heterogeneity was statistically significant, then the random effect model was used. The P value threshold for statistical significance was set at 0.05 for effect sizes. A weighted average of the median survival times with the 95%CI was calculated with Stata 12.0, where the average was weighted with the follow-up period from each study.

## Results

### The included literature and methodological quality

The initial search strategy identified 1647 articles, 1608 of which were excluded after the initial review of their titles and abstracts. After further consideration of the 39 remaining articles, 14 studies [26-39] involving 3,003 patients were finally included in the review according to the inclusion and exclusion criteria. All included articles were observational trials, of which 1,461 patients underwent palliative gastrectomy and 1,542 patients did not received palliative surgery. The characteristics and methodological quality assessment statement are shown in Table 2 and 1, respectively.

**Table 2:** Basic characteristics of trials included in the present study

**Reference**	**Author**	**Year**	**Metastasis**	**Adjuvant chemotherapy**	**With palliative gastrectomy**		**Without palliative gastrectomy**		**1-year survival**		**3-year survival**		**5-year survival**		**Follow-up(month)**	**HR (95%CI)**
					**Patients number**	**Median survival time (month)**	**Ptaients number**	**Median survival time (month)**	**with PG**	**without PG**	**with PG**	**without PG**	**with PG**	**without PG**		
1	Kikuchi S [26]	1998	M1	-	63	12.2	59	5.5	NA	NA	NA	NA	NA	NA	40	0.55 (0.35,0.85)
2	Saidi RF [27]	2006	M0 + M1	±	24	13.2	81	5.5	NA	NA	NA	NA	5%	0	60	0.46 (0.03,7.2)
3	Nazli O [28]	2007	M0	-	21	8	39	3	NA	NA	NA	NA	NA	NA	36	NA
4	Lin SZ [29]	2008	M0 + M1	±	183	NA	206	NA	61.3%	1.3%	8.9%	0	6.2%	0	60	0.61 (0.45,0.82)
5	Lupascu C [30]	2010	M0 + M1	±	30	17.8	45	6.4	NA	NA	NA	NA	NA	NA	30	0.78 (0.06,9.64)
6	Zhang JZ [31]	2011	M0 + M1	-	184	16.4	152	5.5	51.4%	0	NA	NA	NA	NA	60	0.64 (0.49,0.84)
7	Sougioultzis S [32]	2011	M0 + M1	+	218	13.25	93	4	NA	NA	8.1%	3.5%	NA	NA	75	0.08 (0.06,0.13)
8	Kim KH [33]	2011	M1	+	47	15.5	185	9	NA	NA	NA	NA	NA	NA	60	0.65 (0.45,0.94)
9	Chang YR [34]	2011	M1	±	108	12.7	57	11.2	NA	NA	NA	NA	NA	NA	60	0.59 (0.42,0.89)
10	Kokkola A [35]	2012	M1	±	23	10.8	32	5.7	NA	NA	NA	NA	NA	NA	60	0.99 (0.5,1.94)
11	Chen S [36]	2012	M0 + M1	±	392	NA	470	NA	NA	NA	NA	NA	NA	NA	48	0.71 (0.58,0.87)
12	Tokunaga M [37]	2012	M1	±	82	13.1	66	12	NA	NA	NA	NA	NA	NA	60	1.03 (0.65,1.65)
13	Miki Y [38]	2012	M1	±	38	25.6	12	8.7	64.4%	36.7%	36.1%	12.2%	29.4%	0	83	0.27 (0.12,0.59)
14	Dittmar Y [39]	2012	M0 + M1	±	48	15	45	6	NA	NA	NA	NA	NA	NA	60	0.56 (0.32,0.99)
Total publications: 14					1461	14.96*	1542	7.07*								

+: all patients received chemotherapy. -: chemotherapy was not mentioned. ±: a part of patients received chemotherapy.

*: weighted average of median survival time of articles.

### Median survival

Of all included articles, 12 reported median survival times [26-28,30-35,37-39]. In these studies, 885 (58.52%) patients received palliative gastrectomy and 866 (56.16%) patients received other treatments. In the palliative gastrectomy group, the weighted average of the median survival time was 14.96 months (95%CI 14.62–15.29); and in the non-gastrectomy group, the weighted average of the median survival time was 7.07 months (95%CI 6.87–7.27).

### Overall survival

**Figure 1:**
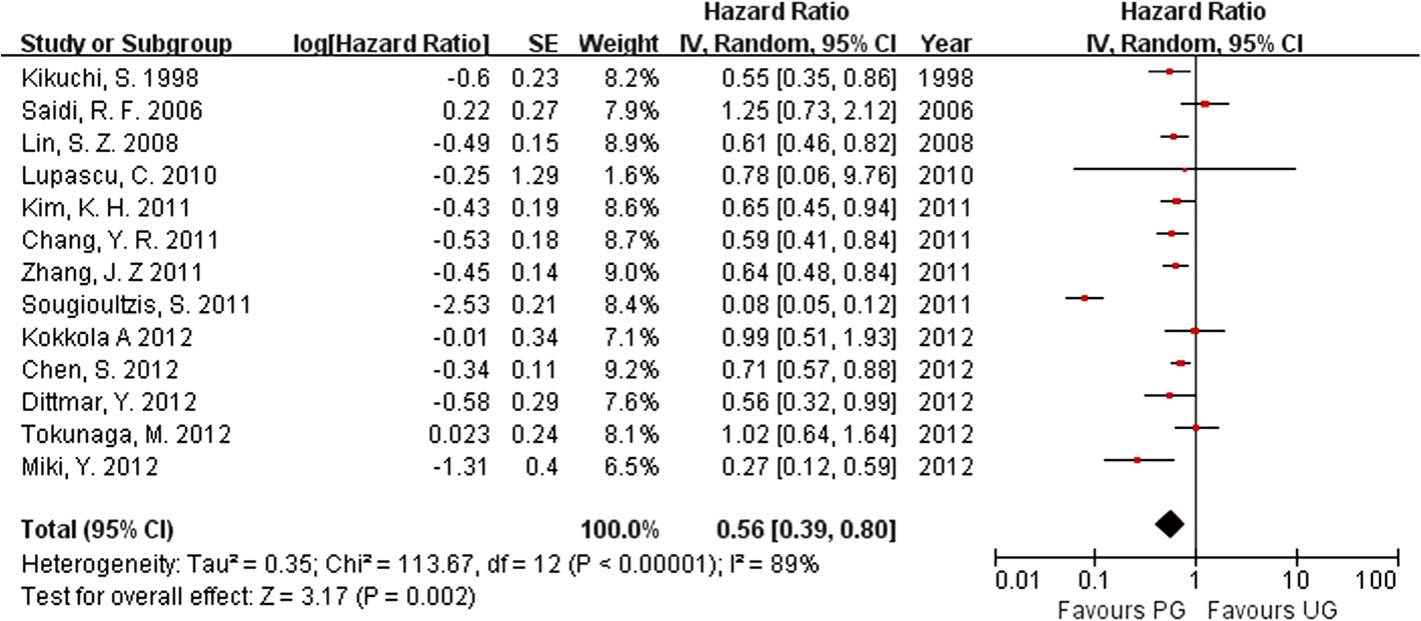
**Hazard ratio for overall survival.** (PG: palliative gastrectomu; NR: no resection).

**Figure 2:**
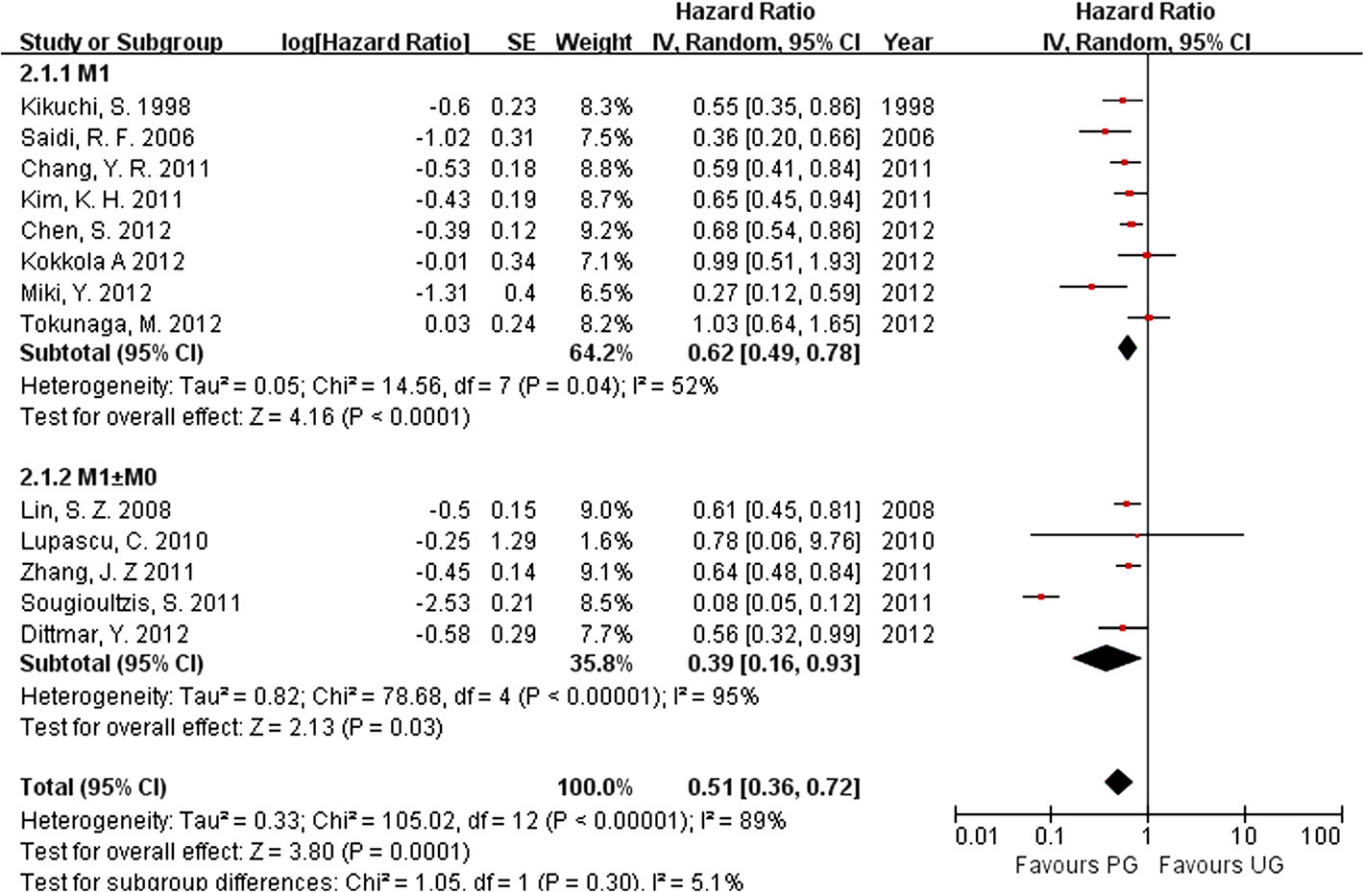
**Hazard ratio for overall survival of subgroups with different M stages.** (PG: palliative gastrectomu; NR: no resection).

Overall survival data were extracted from 13 [26,27,29-39] of the total 14 articles included. Nazli et al. [28] did not report overall survival with in any table or survival curve, so we could not use information for overall survival from their study. In the 13 studies examined, 1440 (98.56%) patients received palliative gastrectomy and 1503 (97.47%) patients received other treatments. Most of the studies demonstrated that palliative gastrectomy improved the long-term survival in patients with incurable GC. The statistical significance of the between-study heterogeneity was examined. The HR for overall survival was 0.56 (95%CI 0.39–0.80; p = 0.0002). The heterogeneity was significant (*P* <0.001, *I*^2^ = 89%; Figure 1). Among these articles, eight [26,27,33-35,37,38] reported stage M1 GC in 1540 patients (51.28%), and five [29-32,39] did not supply detailed data for the 1443 patients (46.72%) investigated in the studies. We analyzed the overall survival rates of the eight studies that clearly reported detailed information about the patients with stage M1 GC. The HR for overall survival in the M1 subgroup was 0.62 (95%CI 0.49–0.78; p < 0.0001); and in the M0 ± M1 subgroup, the HR was 0.39 (95%CI 0.16–0.93; p < 0.0001; Figure 2). Significant between-study heterogeneity was identified in the stage M0 ± M1 GC subgroup (p = 0.03, *I*^2^ = 95%). In the M1 subgroup, the between-study heterogeneity was not highly significant (p = 0.04, *I*^2^ = 52%). Therefore we considered the significant between-study heterogeneity of the articles may be attributable to the M0 ± M1 subgroup, in which the stages were unclear. Palliative gastrectomy showed a tendency to improve the overall survival of patients with advanced GC, especially patients with stage M1 GC.

### Benefit of survival according to different metastatic positions

We researched the concrete metastatic position in patients with stage M1 gastric cancer. Four articles [26,29,36,37] described patients with peritoneal dissemination that received palliative gastrectomy compared to patients without gastrectomy, three articles [29,36,38] reported patients with liver metastasis and two articles [29,36] reported patients with distant lymph node metastasis in detail. The HR of the peritoneal dissemination subgroup was 0.76 (95%CI 0.63–0.92; p = 0.005); the HR of the liver metastasis subgroup was 0.41 (95%CI 0.30–0.55; p < 0.00001); and the HR of the distant lymph-node metastasis subgroup was 0.36 (95%CI 0.23–0.59; p < 0.00001; Table 3). These results show that palliative gastrectomy tends to improve survival in GC patients with peritoneal dissemination, liver metastasis, and distant lymph-node metastasis relative to that of patients receiving other treatments.

**Table 3:** Hazard ratio for overall survival of subgroups

	**No. of studies**	**No. of patients**	**HR (95%CI)**	**p-value**	***I***^**2 **^**(%)**
**Different Metastatic Positions**					
Peritoneal Dissmination [26,29,36,37]	4	832	0.76 (0.63, 0.92)	0.005	68
Liver Metastasis [29,36,38]	3	365	0.41 (0.30, 0.55)	<0.00001	23
Lymph Node Metastasis [29,36]	2	155	0.36 (0.23, 0.59)	<0.00001	90
**Different Regions**					
Western Countries [27,30,32,35,39]	5	639	0.65 (0.58, 0.73)	<0.00001	31
Asian Countries [26,29,31,33,34,36-38]	8	2304	0.23 (0.17, 0.31)	<0.00001	93

### The influence of chemotherapy on palliative gastrectomy

**Figure 3:**
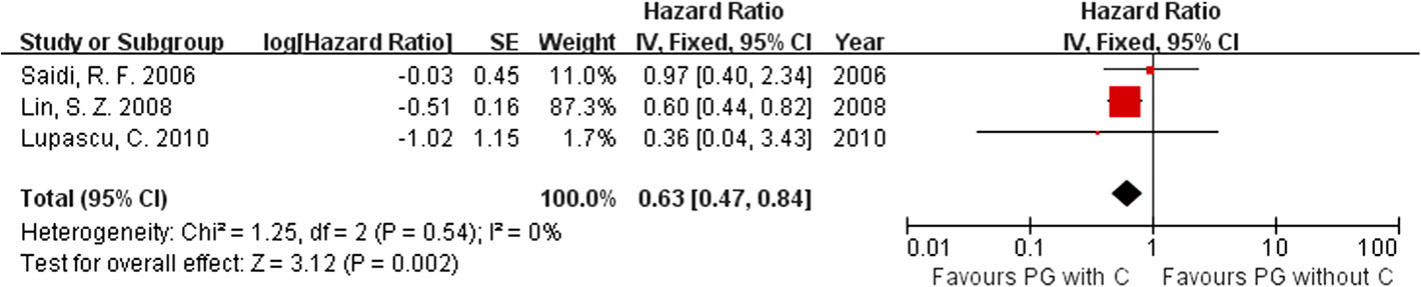
**Hazard ratio for overall survival influenced by chemotherapy.** (C: chemotherapy; CR: combined resection).

Chemotherapy is an important step in treating advanced GC. In all, there were 11 articles [27,29,30,32-39] that mentioned chemotherapy, but only three of them [27,29,30] reported the details on patients with palliative gastrectomy combined with chemotherapy and patients with palliative gastrectomy only. There were 151 patients in the palliative gastrectomy combined with chemotherapy group and 108 patients in the only palliative gastrectomy group. The HR was 0.63 (95%CI 0.47-0.84; p = 0.002; Figure 3). Therefore, chemotherapy may improve the overall survival of patients who receive palliative gastrectomy.

### Palliative gastrectomy with metastasis combined resection

**Figure 4:**
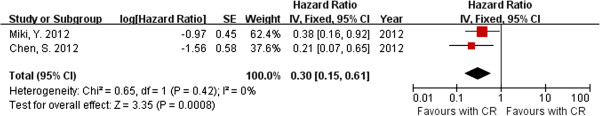
**Hazard ratio for overall survival of patients with liver metastasis received combined hepatic resection or not.** (C: chemotherapy; CR: combined resection).

In all the articles, there were only two studies that described palliative gastrectomy with metastasis combined resection [36,38]. In a study by Chen et al., 25 patients received a combined resection and 29 patients did not. In the study of Miki et al., 25 patients were treated with combined resection and 13 patients were not. In the two studies, the combined resections were all hepatectomies. The HR for overall survival was 0.30 (95%CI 0.15–0.61; p = 0.0008; Figure 4). There was no evidence of statistical heterogeneity (p = 0.42, *I*^2^ = 0%).

## Discussion

In the last 30 years, the patterns of metastasis, recurrence, and survival in patients with GC have changed, and the incidences of GC has decreased worldwide. This phenomenon was promoted by therapies to eradicate approaches of *Helicobacter pylori*[40], improvements in standardized operative procedures and auxiliary instrument, and improvements in the quality of life among different societies, etc. [41]. However, patients with gastric cancer are always in the advanced stage when diagnosed. Recent advances in chemotherapy regimens have improved the survival rates of GC patients with incurable factors. However, whether it is suitable for patients with advanced GC to receive palliative resection is still under debate [42]. Therefore, our study was the first to perform a meta-analysis on palliative resection for patients with incurable advanced GC. The results showed the trend that palliative gastrectomy may improve survival in patients with incurable advanced GC. The impact on the improvement of survival may depend on the position of metastasis, chemotherapy and combined resection of metastasis.

In this study, almost all the articles used median survival time and 1-year, 3-year or 5-year survival rates to assess the effect. Therefore, the overall survival rates extracted from each article were suitable for analysis in this study. The articles that were included in our study all reported patients with incurable advanced GC. However, Kikuchi et al. [26] reported GC with metastasis to the distant peritoneum, so we also included it as an M1 GC. We obtained information about the median survival times of each study from the original articles, and calculated the weighted average values. The results showed that the weighted average of median survival time in patients with palliative gastrectomy was longer than that without palliative resection (14.96 vs. 7.07). Although there was significant heterogeneity, the meta-analysis still showed that palliative gastrectomy tended to improve overall survival rates, with an HR of 0.58 (95%CI 0.48–0.71).

Stage M1 in our study was considered as GC with distant organ metastasis, such as hepatic, peritoneal and distant lymph node metastasis. These had been shown previously to adversely affect survival in several studies [43-45]. Therefore, the characteristic of patients with stage M1 GC was extracted to perform the analysis in our study. These results indicate that GC patients with metastasis who receive palliative gastrectomy may have better overall survival than patients who receive other treatments. In spite of M1 GC showing improvement in survival, fewer included trials may still make obstacles for proving the benefit of palliative gastrectomy in all metastasis types in our study. Furthermore, we investigated palliative gastrectomy combined with resection of metastasis. Sougioultzis et al. [32] reported that there were benefits in palliative gastrectomy for GC patients with distant metastases. Due to limitations of data from the original trials, only combined hepatic resection was in accordance with the selection criteria of our research. Although the efficacy of surgical treatment for hepatic metastasis from GC remains uncertain, palliative gastrectomy may be beneficial for patients with liver metastasis [46] and the results were also the same as in the present study. The results of our meta-analysis confirm that palliative gastrectomy combined with hepatectomy may provide better overall survival than palliative gastrectomy only in patients with advanced GC and liver metastasis.

Several trials reported the function of chemotherapy for patients after palliative gastrectomy. Chemotherapy may be a protective factor for patients with unresectable or metastatic disease, and may offer a comparable survival benefit [47]. Some trials showed there was no survival benefit associated with palliative gastrectomy and recommended chemotherapy [37,42]. In contrast, some articles suggested palliative gastrectomy without chemotherapy was beneficial [48,49]. There were also some trials, such as Lin et al., recommended palliative gastrectomy with chemotherapy to improve the survival rates of patients [29]. The majority of original studies included in the present study reported chemotherapy as used in the trials. We analyzed survival rate of patients that received palliative gastrectomy with or without chemotherapy. The results showed that patients with palliative gastrectomy combined with chemotherapy might have a beneficial survival compared to patients with simple palliative gastrectomy.

Whether a course of treatment is valuable for incurable patients depends on whether it improves their periods of survival and quality of life (QOL). In clinical practice, we must balance the benefits with the risk and costs of surgery, before the decision to treat is taken. Quality of life is an important factor in evaluating the impact of resection, but very few of the articles included in our analysis mentioned. In the present study, we calculated the survival rates to evaluate the efficacy of the treatment, but because we had limited data from only retrospective trials, the quality of life, duration of hospital stay, and costs could not be determined, so the currently available evidence cannot clarify the potential clinical benefits or harms. However, Chang [34] used hospitalization-free survival (HFS) as a parameter to evaluate QOL. The results reported by Chang suggested that palliative gastrectomy may not compromise QOL. However, whether palliative gastrectomy affects QOL, is still contentious, and more research into this tissue is required in the future.

Early diagnosis and prevention strategies are systematically performed in several Asian countries [50], and have been shown to produce a higher rate of early tumor categories and better prognoses among patients with gastric cancer than Europeans and other Western countries. However, many investigations from the countries with the greatest experience and incidence of GC in Asia are published in Asian languages, and are often neither analyzed nor cited. Differences in results may be attributable to different districts. In the present study, eight of the included articles [26,29,31,33,34,36-38] reported in Asian and six [27,28,30,32,35,39] were from Western countries. We analyzed subgroups based on different regions, such as Asian and Western countries, to determine whether they influenced the analysis. Our results showed that palliative gastrectomy may improve survival in both regions, even though relatively clear heterogeneity was observed in the data from Western countries (Table 3). The differences among regions may influence outcomes, but the results of our analysis suggest that the effect is slight. More data from Western countries are required for comparison with Asian data before a final conclusion can be drawn.

**Figure 5:**
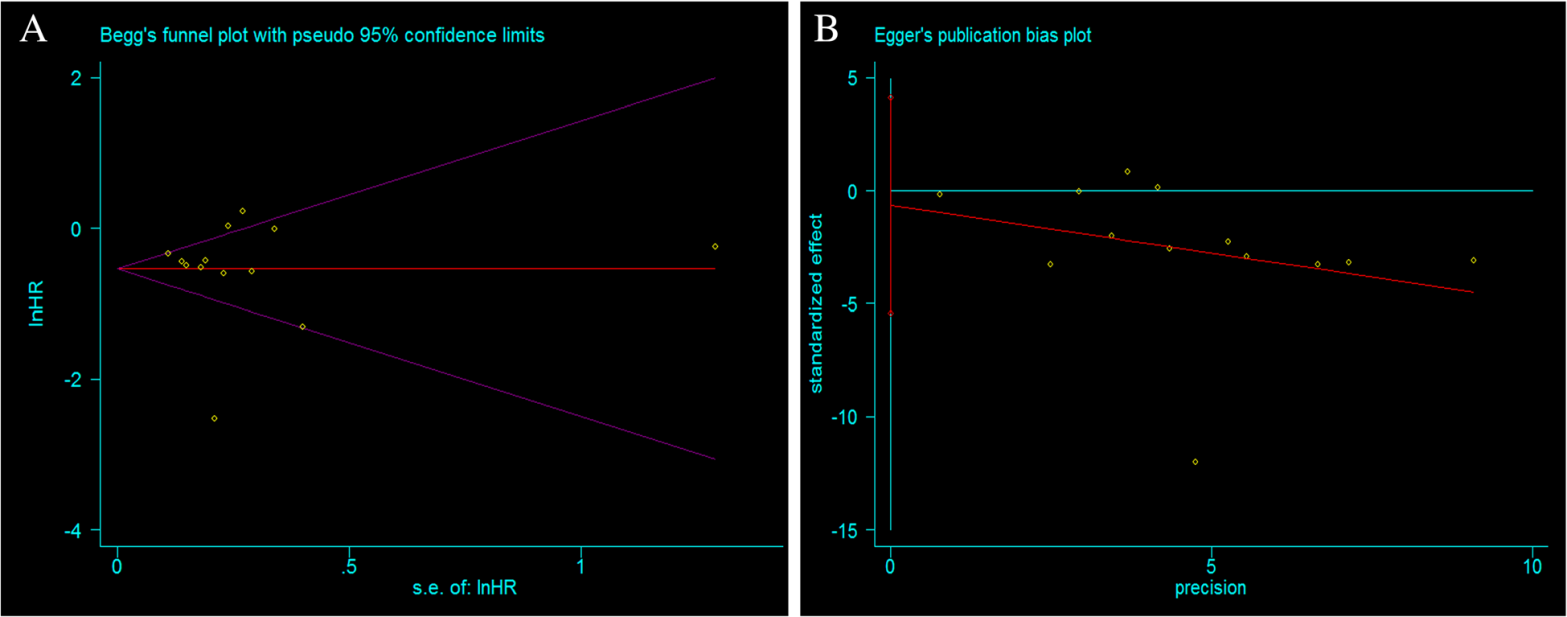
**Test for publication bias. A**. Begger’s test. **B**. Egger’s test.

To date there have been no randomized, controlled trials evaluating the difference between survival of patients managed with or without palliative resection for incurable advanced GC. Therefore, this work was limited to data from retrospective studies and it is difficult to extract strong conclusions from survival data. The patient status and tumor burden at the time of diagnosis may influence the decision as to whether to operate and it may influence the survival advantage after surgery [51]. Only a few trials reported the detailed characteristics of patients before and after surgery [26,29,36-38] (1571 patients, 52.31%), combined resection [36,38] (92 patients, 3.06%), or chemotherapy [27,29,30] (259 patients, 8.62%). We tried to connect with the authors, but few replied. To reduce publication bias, we selected studies carefully and evaluated the trails with the STROBE guidelines. The degree of asymmetry among the individual study results around the combined HR for overall survival in shown in Figure 5. The degree of asymmetry was not statistically significant on Egger’s test (p = 0.177) or Begger’s test (p = 0.855) which means that there was no significant publication bias among the articles included in the present analysis. The selection of patients for the different groups was a problem, because each included study had its own distinct indications and goals, and should be evaluated independently. Without a better understanding of the performance status of the patients selected in the two study groups, selection bias in our analysis cannot be excluded. Furthermore, the quality of articles must be improved in the future. For instance, in the study by Saidi et al., the confidence interval for the median survival time ranged between 4.3 and 28.8 months, which may be attributable to the small sample used in the study (24 patients) or other factors, and sample sizes should be increased to eliminate this effect. Recently, a randomized controlled trial has begun in Japan and Korea to determine the value of gastrectomy performed in patients with advanced GC, and the results are keenly awaited [52]. In the future, well designed and high-quality multicenter clinical trials are still required.

## Conclusion

The present meta-analysis showed that palliative gastrectomy had a statistically significant survival benefit on patients with incurable advanced GC, especially stage M1 GC patients. Survival advantage is longer when chemotherapy was used. For patients with liver metastasis, palliative gastrectomy may provide better survival than with metastasis in other organs. Otherwise, palliative gastrectomy combined with hepatic resection may improve survival.

## Abbreviations

HR: Hazard ratio; GC: Gastric cancer; PG: Palliative gastrectomu; NR: No resection; CR: Combined resection; CI: Confidence intervals; NCCN: National comprehensive cancer network; JGCA: Japanese gastric cancer association; STROBE: Strengthening the reporting of observational studies in epidemiology.

## Competing interests

The authors declare that they have no competing interests.

## Authors’ contributions

JS and YS contributed equally to this work. ZW participated in the conception and design of the study and coordination; JS and YS participated in design of the study, data extraction, article selection and manuscript preparation and interpreted the results in collaboration with YX and HX; JL and XC participated in data extraction, article selection and data extraction; PG performed the statistical analysis and participated in the critical revision of the manuscript. All authors drafted and critically revised the manuscript and approved the final version.

## Pre-publication history

The pre-publication history for this paper can be accessed here:

http://www.biomedcentral.com/1471-2407/13/577/prepub
